# Mobile Fitness and Weight Management Apps: Protocol for a Quality Evaluation

**DOI:** 10.2196/17685

**Published:** 2020-09-24

**Authors:** Madison Milne-Ives, Ching Lam, Michelle van Velthoven, Edward Meinert

**Affiliations:** 1 Digitally Enabled PrevenTative Health Research Group Department of Paediatrics University of Oxford Oxford United Kingdom; 2 Department of Primary Care and Public Health Imperial College London London United Kingdom; 3 Centre for Health Technology Faculty of Health University of Plymouth Plymouth United Kingdom

**Keywords:** mobile apps, telemedicine, smartphone, exercise, weight loss, obesity, physical fitness, fitness trackers

## Abstract

**Background:**

Obesity is a contributing factor for many noncommunicable diseases and a growing problem worldwide. Many mobile apps have been developed to help users improve their fitness and weight management behaviors. However, the speed at which apps are created and updated means that it is important to periodically assess their quality.

**Objective:**

The purpose of this study is to evaluate the quality of fitness and weight management mobile health apps using the Mobile Application Rating Scale (MARS). It will also describe the features of the included apps and compare the results to a previous evaluation conducted in 2015.

**Methods:**

Searches for “fitness,” “weight,” “exercise,” “physical activity,” “diet,” “eat*,” and “food” will be conducted in the Apple App Store and Google Play. Apps that have been updated over the past 5 years will be included. Two reviewers will rate the apps’ quality using the MARS objective and subjective quality subscales. Interrater reliability will also be assessed. Features included in high-quality apps will be assessed, and changes in quality, features, and behavior change techniques made during the past 5 years will be described.

**Results:**

The results will be included in the evaluation paper, which we aim to publish in 2020.

**Conclusions:**

This evaluation will assess the quality of currently available fitness and weight management apps.

**International Registered Report Identifier (IRRID):**

PRR1-10.2196/17685

## Introduction

The number of people who are overweight or obese has tripled since 1975, and in 2016, 40% of adults were overweight [1]. Obesity is a major concern for public health because it increases the likelihood of many preventable diseases (eg, cardiovascular diseases, diabetes, osteoarthritis, and some cancers) and places an economic burden on the health care system [1,2]. However, obesity is largely a preventable condition [3]. Increasing physical activity and eating healthier foods can help people to manage their weight, and thus reduce the consequences of chronic and preventable diseases [1,4].

Since the first smartphone was released in 2008, digital technologies have become an increasingly common and popular way for people to change their health behaviors [5]. Mobile apps are a useful platform to provide behavioral interventions to improve fitness and weight because of the widespread use of smartphones and the large number of mobile health apps available [6]. Some evidence of the acceptability and effectiveness of mobile health apps for increasing physical activity, improving eating behaviors, and reducing weight has been found [4,7], but additional evidence is still needed to strengthen the conclusion that mobile apps are effective at improving health outcomes in particular [8-10]. The mixed body of evidence of effectiveness might be due to the fact that higher engagement has been found to be related to increased adherence to the app and weight loss [4]. Therefore, it is important to assess the quality of mobile fitness and weight management apps because quality is likely to influence engagement, which will affect the effectiveness of these apps at changing behavior and causing weight loss.

Evaluations have previously examined the quality of mobile weight management apps [11,12]. However, testing for these evaluations was conducted in August 2014 [12] and the beginning of 2015 [11]. Given the rate at which apps are being developed and updated [13], evaluations should be conducted every couple of years to assess the quality of currently popular mobile fitness and weight management apps. Evaluations can then be compared to track whether there have been any changes in the quality or features of popular apps over time. Both of the previous evaluations assessed popular iOS and Android apps. Bardus et al [11] evaluated the apps using the MARS subscales and found that the overall quality of apps was moderate, while Chen et al [12] focused on Australian apps and concluded that the overall quality was suboptimal. The authors of both reviews also examined the behavior change techniques (BCTs) [14] that were incorporated in the apps. Experts have established a theory-based taxonomy of these BCTs to aid identification and evaluation of the key components of behavioral interventions [14]. The review of Australian apps found a general lack of BCTs [12], while self-monitoring of behavior and outcomes, goal setting for behavior and outcomes, and feedback on behavior and outcomes were identified as the most common BCTs in Bardus et al’s review [11].

In their evaluation, Bardus et al [11] concluded that improvements could be made to app quality by focusing on information quality and evidence-based content. A similar, more current evaluation will provide an update on both the quality of mobile fitness and weight management apps and which BCTs are included in them. This will allow an assessment of whether and how mobile fitness and weight management apps have changed in the past 5 years. There are also improvements that can be made to the previous review methodologies: Bardus et al’s evaluation [11] only included apps that focused on a combination of diet and physical activity interventions and had a version available in both the Apple App Store and Google Play, while Chen et al’s review [12] did not use a standardized overall measure of app quality (the MARS measure was not yet published [15]). The proposed evaluation will broaden that scope by also including apps that focus on only diet or physical activity and apps available only in the App Store or Google Play, as this will better represent the broad range of apps that people use to improve their fitness and weight management. Additionally, our evaluation will compare our findings with Bardus et al’s [11] to examine how the general state of app quality and the inclusion of BCTs have changed in the past 5 years.

Therefore, this evaluation will be focused on three main research questions. First, what is the objective and subjective quality of various Apple and Android mobile fitness and weight management apps, as measured by the Mobile Application Rating Scale? Second, what are the features most commonly associated with high-quality apps? Third, how have the quality and included BCTs of popular apps changed since 2015?

## Methods

### Overview

We will use the PRISMA-P (Preferred Reporting Items for Systematic Reviews and Meta-Analyses Protocols) guidelines [16] to guide the search and selection of apps for evaluation. This evaluation will be composed of an app search, app selection, data extraction, data analysis, and data synthesis.

### Search Strategy

We will search the Apple App Store and Google Play to identify current popular mobile fitness and weight management apps. We will search each of the following keywords: “fitness,” “weight,” “exercise,” “physical activity,” “diet,” “eat*,” and “food.” These were chosen based on commonly used terms in the literature [4]. The search results will be filtered by popularity (based on the stores’ display algorithms) and the 100 most popular apps from each search will be screened. This will ensure that the apps being evaluated are the ones that are most used and will limit the number of apps to be evaluated.

### Eligibility

#### Inclusion Criteria

We will include popular apps that aim to improve health-related fitness and weight management behaviors, specifically diet or physical activity, and that target the general population. This will include apps designed for any age group, from children to older adults.

#### Exclusion Criteria

We will exclude duplicates (if an app is available for both iOS and Android operating systems, we will include the iOS version only) and apps that are not in English. We will also exclude apps that have not been updated in the past 5 years [17]. We will exclude any apps that do not provide dietary or physical activity behavioral interventions that aim to improve general health and fitness or weight management. Therefore, recipe apps, athletic training apps, and apps that are focused on behaviors that are not health-related (like looking younger) will be excluded. Apps that are focused on specific populations (eg, people with specific diseases or pregnant women) will also be excluded.

### Screening and App Selection

All of the apps found in the search will be recorded in an Excel document (Microsoft Corp) and duplicates will be removed, including Android apps that also have an iOS version. Preliminary screening by two independent reviewers will determine initial eligibility for the evaluation using the information provided in the app summaries on the App Store and Google Play. Apps that are deemed eligible will be downloaded. Apps that, upon closer examination, do not meet the inclusion criteria will also be excluded. Any disagreements between the reviewers will be discussed and, if necessary, settled by a third reviewer. All of the apps identified as being eligible for inclusion will be reviewed. A PRISMA flow diagram will be used to record the details of the search, screening, and selection processes so that the evaluation can be reproduced.

### Data Extraction

The apps will be tested and evaluated by two independent reviewers. Each app will be used for at least half an hour before being rated using the MARS scales [15,18]. The reviewers will also extract general information about the app as well as its features (eg, how it tracks behaviors or outcomes; if it provides notifications, feedback, or information) and any BCTs [14] that are included. The items to be extracted are summarized in Textbox 1.

**Data that will be extracted from the apps.**

**General information**

- Year of development
- Platform (iOS, Android, etc)
- Developers
- Target population
- Target behavior change (eg, diet, step count, exercise)
- Cost
- Number of downloads
- App Store or Play Store rating

**Features**

- App features (eg, notifications, tracking, feedback)
- Behavior change techniques (BCT Taxonomy v1 [14])

**Quality (all are Mobile Application Rating Scale subscales [15])**

- Engagement
- Functionality
- Visual esthetics
- Information quality
- Subjective quality

### Data Analysis and Synthesis

The Mobile Application Rating Scale will be used to evaluate the quality of the included apps. Both reviewers will complete a training exercise in the MARS (which will be requested from the corresponding author of the MARS development paper) before conducting the evaluation [15]. The MARS has a total of 23 items split into 5 different subscales: engagement, functionality, esthetics, information, and subjective quality. Each item is rated on a 5-point Likert scale. The overall score is calculated by averaging the mean scores of the subscales, with objective and subjective ratings kept separate [15]. Interrater reliability of the two reviewers will also be assessed.

The objective and subjective scores of the various apps will be compared to determine which apps have the highest quality. The features and BCTs of the 20 highest-rated apps will also be examined, to determine which features are associated with the highest-quality apps.

## Results

The results will be included in the evaluation paper, which we aim to publish in 2020.

## Discussion

A systematic evaluation of mobile fitness and weight management apps will provide a clearer assessment of their quality. There are many fitness and weight management apps to choose from, and star rating systems have not been found to be strongly correlated with the MARS measure of app quality [15]. An evaluation of these apps will help consumers choose higher quality apps and will contribute to the literature on and the improvement of mobile health behavior change apps by examining which features and BCTs [14] are common in high-quality apps. These results will be compared to a previous evaluation of the quality of and BCTs included in mobile weight management apps and describe whether and how popular apps have changed since 2015 [11]. Based on the data, this section will compare the included apps, discuss the limitations of the evaluation, and consider important directions for future research. One limitation that can already be identified is the use of only two reviewers. Given the significant time requirements to evaluate each app in depth, it is only feasible to use two reviewers; although the reviewers will work independently, it is possible that they will be biased in a way that might not be identified.

## Abbreviations

BCT: behavior change technique
MARS: Mobile Application Rating Scale
PRISMA-P: Preferred Reporting Items for Systematic Reviews and Meta-Analyses Protocols
